# ONECUT2 overexpression promotes RAS-driven lung adenocarcinoma progression

**DOI:** 10.1038/s41598-019-56277-2

**Published:** 2019-12-27

**Authors:** Qingyang Ma, Kai Wu, Hui Li, Huichun Li, Yufei Zhu, Guohong Hu, Landian Hu, Xiangyin Kong

**Affiliations:** 1 CAS Key Laboratory of Tissue Microenvironment and Tumor, Shanghai Institute of Nutrition and Health, Shanghai Institutes for Biological Sciences, Chinese Academy of Sciences, Shanghai, China; 2 University of Chinese Academy of Sciences, Beijing, China; 3 School of Life Science and Technology, ShanghaiTech University, Shanghai, China

**Keywords:** Non-small-cell lung cancer, Cancer epigenetics

## Abstract

Aberrant differentiation, driven by activation of normally silent tissue-specific genes, results in a switch of cell identity and often leads to cancer progression. The underlying genetic and epigenetic events are largely unexplored. Here, we report ectopic activation of the hepatobiliary-, intestinal- and neural-specific gene one cut homeobox 2 (*ONECUT2)* in various subtypes of lung cancer. ONECUT2 expression was associated with poor prognosis of RAS-driven lung adenocarcinoma. ONECUT2 overexpression promoted the malignant growth and invasion of A549 lung cancer cells *in vitro*, as well as xenograft tumorigenesis and bone metastases of these cells *in vivo*. Integrative transcriptomics and epigenomics analyses suggested that ONECUT2 promoted the trans-differentiation of lung cancer cells by preferentially targeting and regulating the activity of bivalent chromatin domains through modulating Polycomb Repressive Complex 2 (PRC2) occupancy. Our findings demonstrate that ONECUT2 is a lineage-specific and context-dependent oncogene in lung adenocarcinoma and suggest that ONECUT2 is a potential therapeutic target for these tumors.

## Introduction

Lung cancer encompasses a heterogenous group of malignancy. Adenocarcinoma, squamous cell carcinoma and small cell lung cancer have distinct cell of origin, in which pulmonary epithelial differentiation are maintained by a series of lineage-specific transcription factors and pathways^1^. Lineage transition from adenocarcinoma to squamous cell carcinoma, as well as from non-small cell lung cancer to small cell lung cancer have been reported^2,3^. Besides, trans-differentiation could lead to loss of pulmonary identity, which might be associated with disease progression and poor prognosis. Loss of Nkx 2-1, a factor essential for lung morphogenesis and alveolar cell differentiation, results in gastric differentiation and dissemination of tumor with *KRAS* and *TP53* mutations^4–6^. The contribution of other lineage-committing factors to aberrant differentiation in lung cancer malignancy are not well understood.

Cell identity is stabilized by Polycomb Repressive Complex 2 (PRC2) by maintaining gene silencing^7^. Both gain-of-function and loss-of function mutations of PRC2 occurs in cancer and a number of studies have demonstrated that PRC2 has both oncogenic and tumor-suppressive roles in cancer^7^. Moreover, the tumor suppressive role of PRC2 depends on mutation status of RAS signaling pathway and other oncogenic alterations. Loss of SUZ12 collaborates with NF1 mutation to amplify RAS-driven transcription in peripheral nerve sheath tumors^8^. *Eed* deletion accelerates tumor formation in a mutant *KRas*-driven, *Trp53*-null mouse model of non-small-cell lung cancer^9^. However, the transcription factors overruling context-dependent PRC2-mediated repression in cancer remain unexplored.

One cut homeobox 2 (ONECUT2) is a transcription factor with two DNA-binding domains-a Homeobox domain and a Cut domain. In human and mice, ONECUT2 is highly expressed in hepatobiliary tissues, small intestine and the nervous system. Together with its paralog ONECUT1, ONECUT2 redundantly regulates fate specification of pancreatic cells^10–12^ and fate determination of retinal horizontal cells^13–16^. Interestingly, ONECUT2, but not ONECUT1, is overexpressed in prostate cancer and lung cancer^17^. Of note, ONECUT2 is a driver of neuroendocrine prostate cancer^18^. More importantly, ONECUT2 is a targetable master regulator of metastatic castration-resistant prostate cancer^19^. However, the role of ONECUT2 in lung cancer progression is unknown.

Here, we report that *ONECUT2* is a lineage-specific and context-dependent oncogene in lung adenocarcinoma. Targeted manipulation of histone methylation orchestrated by the oncogenic transcriptional regulation of ONECUT2 contributes to the epigenetic reprogramming, aggressive behavior and metastasis of RAS-driven lung cancer.

## Results

### ONECUT2 is aberrantly activated in lung cancer

**Figure 1:**
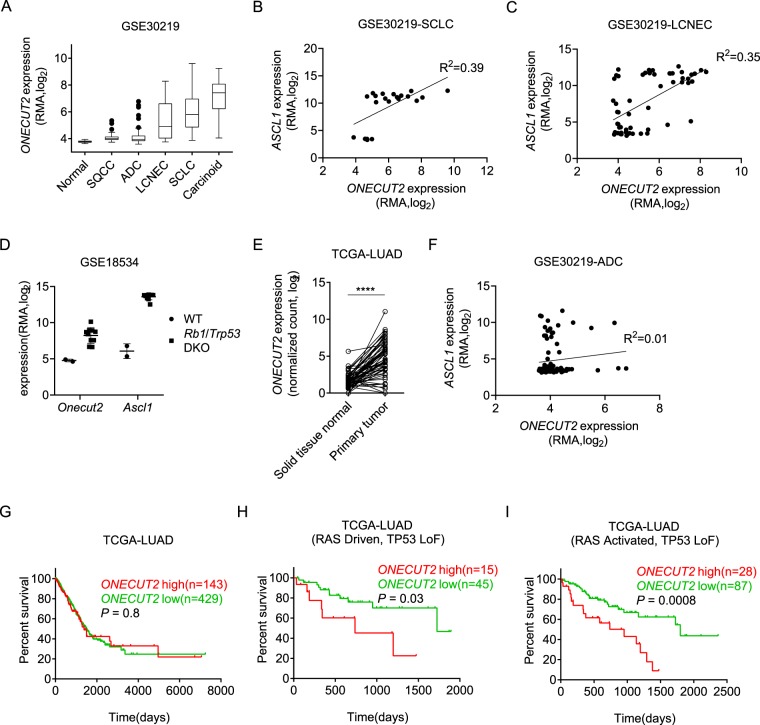
ONECUT2 is aberrantly expressed in lung cancer. (**A**) ONECUT2 expression in various lung cancer subtypes from dataset GSE30219. Data are presented as a Tukey box plot. SQCC: squamous cell carcinoma. (**B**) Scatter plot of ASCL1 and ONECUT2 expression of SCLC samples from GSE30219. (**C**) Scatter plot of ASCL1 and ONECUT2 expression of LCNEC samples from GSE30219. (**D**) Dot plot of Onecut2 and Ascl1 expression in wide type (WT) and *Rb1/Trp53* double knockout (*Rb1/Trp53* DKO) mice from dataset GSE18534. Error bars indicate mean ± SD. (**E**) Dot plot of ONECUT2 expression of 58 ADC samples and paired normal tissues from TCGA (****p < 0.0001, paired t-test). (**F**) Scatter plot of ASCL1 and ONECUT2 expression of ADC samples from GSE30219. (**G**) Overall survival analysis of ADC patients from TCGA-LUAD stratified by the upper quantile expression level of ONECUT2. (**H,I**) Overall survival analysis of RAS-driven (**H**) ADC patients or patients with oncogenic alterations in RTK/RAS/BRAF pathway (**I**) in the presence of TP53 loss of function alterations from TCGA-LUAD stratified by upper quantile expression level of ONECUT2.

We first analyzed ONECUT2 expression across various subtypes of lung cancer using publicly available dataset GSE30219^20^. ONECUT2 was highly expressed in small cell lung cancer (SCLC), large cell neuroendocrine lung cancer (LCNEC) and lung carcinoid tumor (Carcinoid), a subtype with low-grade malignancy **(**Fig. 1A**)**. Since these three subtypes are all manifested by neuroendocrine differentiation, we suspected that ONECUT2 was a neuroendocrine differentiation gene in lung tumors. Indeed, ONECUT2 expression correlated with ASCL1, a well-defined master regulator of neuroendocrine differentiation, in SCLC and LCNEC **(**Fig. 1B,C, Supplementary Fig. 1A**)**. Moreover, both ONECUT2 and ASCL1 are significantly upregulated in a mouse model of small cell lung cancer where *Rb1* and *Trp53* are both knocked out **(**Fig. 1D**)**, indicating a shared regulation. Considering ASCL1 had been proved to be a lineage-specific oncogene in high-grade neuroendocrine lung cancer and high expression of ONECUT2 in lung carcinoid tumor, we believed that ONECUT2 was unlikely a driver oncogene in lung tumor with neuroendocrine differentiation.

Besides SCLC, LCNEC and carcinoid, ONECUT2 was also highly expressed in a subset of lung adenocarcinoma (ADC) **(**Fig. 1A**)**, the most frequent subtype of lung cancer. Analysis of TCGA-LUAD dataset indicated that ONECUT2 expression was elevated in cancerous tissue compared with paired adjacent normal tissues **(**Fig. 1E**)**. Moreover, tissue microarray of lung adenocarcinoma and matched adjacent normal specimen showed that ONECUT2 was stained in 21 out of 75 tumor samples (nuclear positivity of 28%) **(**Supplementary Fig. 1B**)**. While ASCL1 was also overexpressed in a subset of lung adenocarcinoma, the expression of ONECUT2 and ASCL1 were barely correlated **(**Fig. 1F, Supplementary Fig. 1C**)**. Furthermore, neither ASCL1 nor ONECUT2 overexpression was associated with poor prognosis in ADC **(**Fig. 1G, Supplementary Fig. 1D–F**)**. By integrated analysis of gene expression and genomic alteration profiles of TCGA ADC samples, we discovered that ONECUT2 overexpression was significantly associated with shorter overall survival in RAS-driven ADC patients **(**Fig. 1H**)** or patients with oncogenic alterations in the RAS signaling pathway (RAS activated) **(**Fig. 1I**)** in the presence of TP53 loss of function alterations (See the definition of RAS-driven and RAS-activated tumor in the Methods section). However, ASCL1 overexpression was not associated with overall survival in the corresponding genomic alteration context **(**Supplementary Fig. 1G,H**)**. These data implied that ONECUT2 was a context-dependent prognostic factor and oncogene in lung adenocarcinoma.

### Overexpression of ONECUT2 promotes malignant growth and invasion of A549 cells *in vitro*

**Figure 2:**
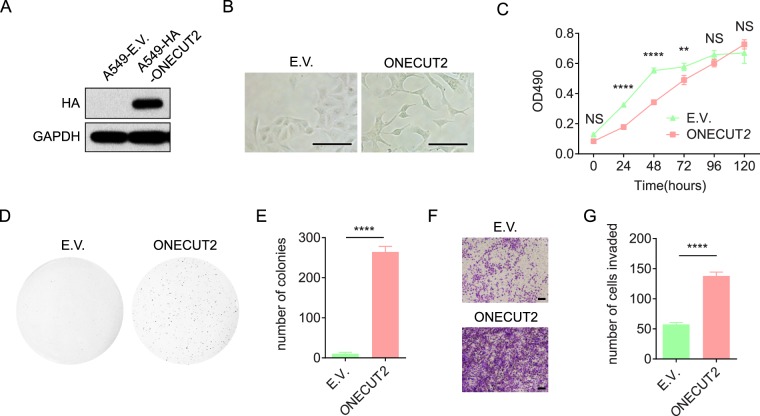
ONECUT2 overexpression enhances malignant growth, migration, invasion and adhesion of A549 cells *in vitro*. (**A**)Western Blot of whole cell lysates from A549-ONECUT2 and A549-E.V. cells using antibodies against HA and GAPDH. The full-length blot is presented in Supplementary Fig. 5. (**B**) Light microscopic visualization of A549-ONECUT2 and A549-E.V. cells; scale bar: 50 µm. (**C)** Two-dimensional cell proliferation assay of A549-ONECUT2 and A549-E.V. cells. Proliferation is measured as absorbance at 490 nm (OD490). Error bars indicate mean ± SD. (**D**) Soft agar colony formation assay of A549-ONECUT2 and A549-E.V. cells. Images shown are representatives of three independent experiments. (**E**) Quantification of colonies in soft agar colony formation assay. Error bars indicate mean ± SD (****p < 0.0001, Student’s t-test). (**F**) In *vitro* two chamber transwell assay of A549-ONECUT2 and A549-E.V. cells. Images shown are representative of three independent experiments. (**G**) Quantification of invading cells in the transwell assay. Error bars indicate mean ± SD (****p < 0.0001, Student’s t-test).

We hypothesized that ONECUT2 promoted RAS-driven lung adenocarcinoma malignancy. We chose A549, a human lung adenocarcinoma cell line with homozygous KRAS^G12S^ mutation, to investigate the role of ONECUT2 in lung cancer progression. Retroviral transduction of the *ONECUT2* gene resulted in A549 cells with stable overexpression of ONECUT2 (A549-ONECUT2) **(**Fig. 2A**)**. A549 cells showed profound morphological changes upon ONECUT2 overexpression, with elongated cell bodies, extended dendrites and loosened cell-cell contacts **(**Fig. 2B**)**. Compared with A549 cells transduced with the empty vector (A549-E.V. cells), A549-ONECUT2 cells showed a lower rate of proliferation in low density two-dimensional cell culture, but maintained a high proliferation rate even when confluent, suggesting a loss of contact inhibition **(**Fig. 2C**)**. Indeed, A549-ONECUT2 cells formed more colonies in soft agar **(**Fig. 2D,E**)**, indicating a gain of malignant growth.

Next, we examined the effect of ONECUT2 overexpression on the migratory behavior of A549 cells. As expected, overexpression of ONECUT2 significantly promoted the migration and invasion of A549 cells, as demonstrated by a wound healing assay **(**Supplementary Fig. 2A**)** and an *in vitro* two-chamber Matrigel transwell invasion assay **(**Fig. 2F,G**)**, respectively. Because loss of cell-cell contact and establishment of cell-substrate interaction are critical steps in the invasion of cancer cells, we further tested the changes in cell-matrix contact in response to ONECUT2 overexpression. As illustrated in Supplementary Fig. 2B, A549-ONECUT2 cells adhered much more readily to type I collagen, type IV collagen, and Matrigel than A549-E.V. cells, suggesting a remodeling of the cell-matrix interaction. Overall, we concluded that overexpression of ONECUT2 promoted the malignant growth, migration, invasion and adhesion of A549 lung cancer cells.

### Overexpression of ONECUT2 promotes xenograft tumorigenesis and bone metastasis of A549 cells *in vivo*

**Figure 3:**
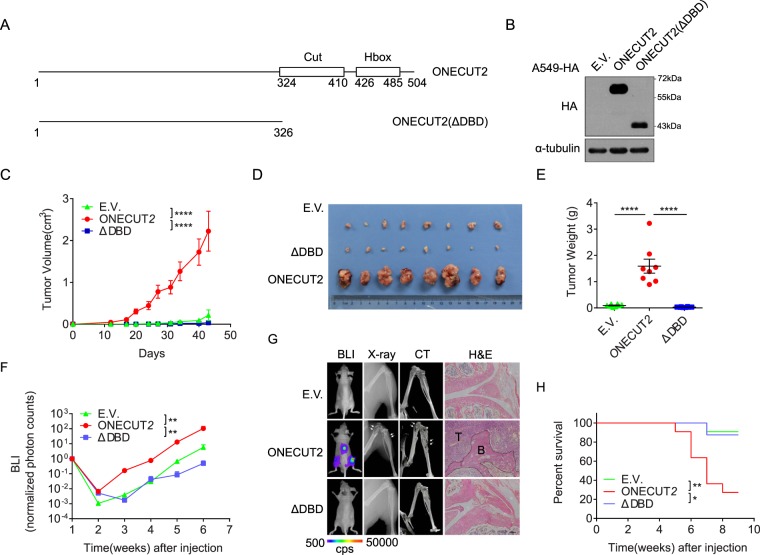
ONECUT2 promotes the xenograft tumorigenesis and bone metastasis of A549 cells *in vivo*. (**A**) Schematic view of ONECUT2 and its ΔDBD mutant. Cut domain and Homeobox domain along with their position are shown. (**B**) Western Blot of whole cell lysates from A549-ONECUT2, A549-E.V. and A549-ONECUT2(ΔDBD) cells using antibodies against HA and α-tubulin. The full-length blot is presented in Supplementary Fig. 5. (**C**) Quantification of xenograft tumor volume. Error bars indicate mean ± SD (****p < 0.0001, t-test). (**D**) Image of isolated tumor masses. (**E**) Scatter dot plot of tumor weight at endpoint. Mean of each group are shown (****p < 0.0001, t-test). (**F**) Quantification of bioluminescence imaging (BLI) of bone metastasis burden (*p < 0.01, ns: not significant, t-test). (**G**) *In vivo* analysis of bone metastasis. Shown are representative mice at 4 weeks after cancer cell implantation; BLI, X-ray, micro-CT and hematoxylin and eosin (**H/E**) staining of animal hind limbs 6 weeks after cancer cell implantation. Areas circled by dotted lines denote tumor regions, and arrows indicate the regions of bone damage. (**H**) Overall survival curves of mice injected with different cell lines (*p < 0.01, **p < 0.001, log rank test).

We further investigated the function of ONECUT2 overexpression *in vivo*. To this end, we made a truncated mutant of ONECUT2 lacking both the Cut and Homeobox DNA-binding domains – designated ONECUT2(ΔDBD) – and transduced A549 cells **(**Fig. 3A,B**)**. We inoculated A549-ONECUT2, A549-ONECUT2(ΔDBD) and A549-E.V. cells subcutaneously at the base of the left forelimb in nude mice. A549-ONECUT2 cells, compared with A549-E.V. cells and A549-ONECUT2(ΔDBD) cells, gave rise to much larger tumors (as measured by both tumor mass and volume) over a period of 40 days **(**Fig. 3C–E**)**. The ΔDBD mutant did not confer A549 with greater tumorigenesis potential, suggesting that DNA binding was essential for ONECUT2 function.

Next, we analyzed the role of ONECUT2 in metastasis by injecting A549-ONECUT2, A549-ONECUT2(ΔDBD) and control A549-E.V. cells intracardially into nude mice. Overexpression of ONECUT2 but not its ΔDBD mutant significantly enhanced bone metastasis of A549 cells as revealed by bioluminescent imaging (BLI), X-ray analysis, micro-CT scan and histological examination **(**Fig. 3F,G, Supplementary Fig. 3**)**. Tumor cells colonized exclusively the spine and hind limbs. The metastases manifested as severe osteolytic bone lesions and massive bone destruction, as demonstrated by X-ray and CT scans **(**Fig. 3G**)**. Hematoxylin and eosin staining of the metastatic sites showed massive invasion of tumor cells and distortion of normal bone structure. Importantly, mice bearing tumors composed of A549-ONECUT2 cells following intracardial injection had a significantly shorter lifespan than those bearing tumors composed of A549-ONECUT2(ΔDBD) or control cells **(**Fig. 3H**)**. These data collectively indicated that ONECUT2 overexpression enhanced osteolytic bone metastasis *in vivo* and that this effect was dependent on ONECUT2’s DNA binding domains.

### Overexpression of ONECUT2 activates oncogenic pathways and lineage-specific genes

**Figure 4:**
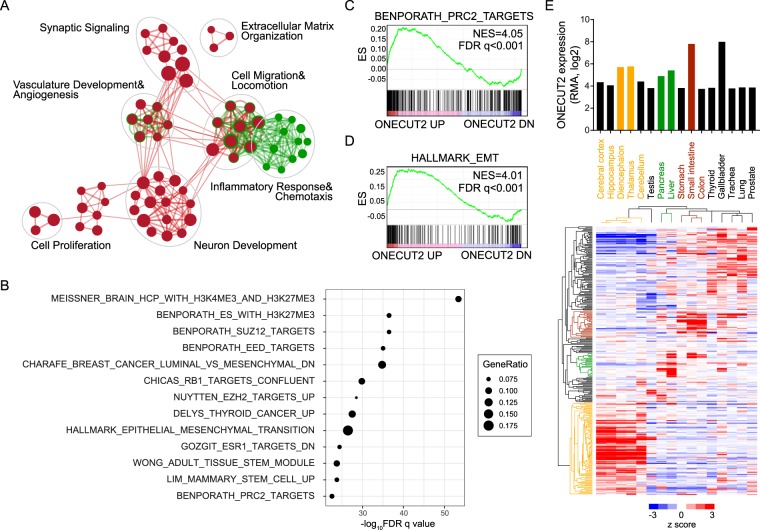
ONECUT2 overexpression activates lineage-specific genes and oncogenic pathways. (**A**) A total of 1285 DEGs were subjected to DAVID GO analysis. An enrichment map was constructed by using Cytoscape with the Enrichment Map plugin. Red nodes represent enriched GO biological pathways of upregulated genes, and green nodes and rings represent enriched GO biological pathways of downregulated genes (p < 1e-05, FDR q < 1e-04, overlap cutoff > 0.68). Nodes and ring sizes are proportional to the total number of genes in each pathway. Edge thickness represents the number of overlapping genes between nodes. GO pathways of similar functions are clustered together and are marked with circles and labels. (**B**) Top 10 gene sets in MSigDB that overlapped with genes upregulated by ONECUT2 overexpression. (**C,D**) GSEA of PRC2 targets (**C**) and EMT(**D**) gene signature on ONECUT2 DEGs. Genes were ordered by expression fold changes by ONECUT2 overexpression in descending order from left to right. (**E**) Lineage-restricted expression pattern of ONECUT2-upregulated genes. Hierarchy clustering of expression of ONECUT2-upregulated genes across multiple normal tissues, alongside with expression of ONECUT2.

To understand the molecular events underlying ONECUT2, we set out to investigate the transcriptional targets modulated by ONECUT2 using A549-ONECUT2 and A549-ONECUT2(ΔDBD) cells. By RNA-Seq, we identified 667 genes upregulated and 618 genes downregulated in A549-ONECUT2 cells relative to A549-ONECUT2(ΔDBD) cells **(**Supplementary Table 1**)**. We used DAVID to analyze the Gene Ontology biological pathways significantly enriched in the differentially expressed genes (DEGs). Enrichment map categorized enriched pathways with significant overlapping genes into several functional clusters **(**Fig. 4A**)**. Specifically, genes in neuron development, cell locomotion and migration, angiogenesis, proliferation and extracellular matrix organization pathways were enriched in upregulated genes. By contrast, downregulated genes were mainly associated with inflammatory responses and chemotaxis.

We further investigated the protein-protein interaction networks of the DEG products using the STRING database. The subnetworks of ONECUT2 downstream targets are shown in Supplementary Fig. 4A–E. We found predominant and dense interaction networks of genes engaged in neural differentiation, including the well-characterized neuroendocrine regulator somatostatin (SST) and its receptor, as well as CXCR4 and CXCR7, two chemokine receptors well-implicated in cancer metastasis **(**Supplementary Fig. 4A**)**. We also discovered a subnetwork with repressed E-cadherin (CDH1) and activated OB-cadherin (CDH11) at the center, indicating a “cadherin switch” in response to ONECUT2 overexpression **(**Supplementary Fig. 4B**)**. Another noteworthy network was characterized by increased matrix metalloproteinase 1 (MMP1) expression and modulation of several extracellular matrix proteins, thus indicating a potential role of ONECUT2 in cell-matrix interaction and remodeling **(**Supplementary Fig. 4C**)**. Wnt signaling pathways were also significantly perturbed, as evidenced by altered expression of several WNT modulators, such as CER1, GLI3, HEY1 and DKK2 **(**Supplementary Fig. 4D**)**. Finally, the tyrosine kinase receptor gene PDGFRA was significantly upregulated along with its genetic and physical neighbors, further underscoring ONECUT2’s oncogenicity **(**Supplementary Fig. 4C**)**. These data suggested that ONECUT2 overexpression initiated transcriptional activation programs enhancing the malignancy of lung adenocarcinoma cancer cells.

By comparing DEGs with gene sets in MSigDB, we found genes upregulated by ONECUT2 overexpression significantly overlapped with genes bearing bivalent histone marks, PRC2 targets and hallmark genes of epithelial-to-mesenchymal transition **(**Fig. 4B–D**)**. Moreover, consistent with the lineage-restricted expression pattern of ONECUT2, ONECUT2 activated genes specifically expressed in neural, hepatobiliary and intestinal tissues, suggesting the activation of trans-differentiation programs in ONECUT2-overexpressed lung cancer cells **(**Fig. 4E**)**.

### ONECUT2 preferentially occupies and modulates the activity of bivalent promoters

**Figure 5:**
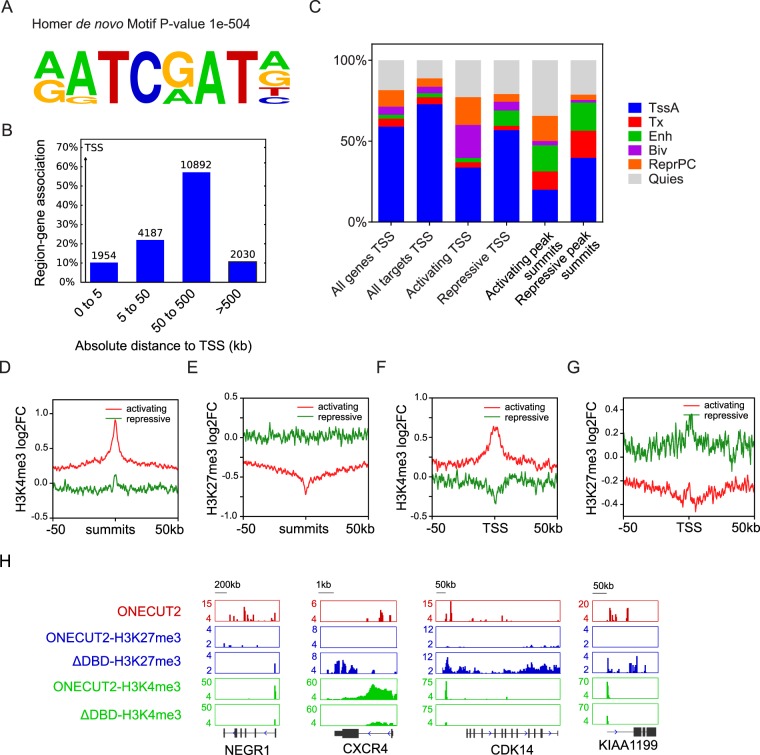
ONECUT2 binds and modulates the activity of bivalent chromatin. (**A**) Homer motif analysis of high confidence ONECUT2 peaks. (**B**) GREAT analysis of high confidence ONECUT2 peaks shows distribution of gene-region assignments around TSS. (**C)** Percentage plot of Roadmap A549 cell chromatin states around activated and repressed ONECUT2 target gene TSS, as well as summits of activating and repressive peaks. Chromatin state around the TSSs of all protein-coding genes and all ONECUT2 target genes are shown as control. (**D,E**) Plot of average H3K4me3 **(D**) and H3K27me3 (**E**) log_2_ fold change by ChIP-Seq (A549-ONECUT2 versus ΔDBD) around activating and repressive ONECUT2 peaks. (**F,G**) Plot of average H3K4me3 (**F**) and H3K27me3 (**G**) log_2_ fold change by ChIP-Seq (A549-ONECUT2 versus ΔDBD) around TSS of genes associated with activating and repressive ONECUT2 peaks. (K) Representative illustration of H3K4me3 and H3K27me3 signals around four selected loci alongside ONECUT2 binding signals in A549-ONECUT2 cells.

To study the mechanism of transcription regulation by ONECUT2, we used an anti-hemagglutinin (anti-HA) antibody to isolate protein-DNA complex in A549-ONECUT2 cells and high-throughput sequencing (ChIP-Seq) to reveal its DNA binding pattern. We identified over 10800 high confidence binding peaks (*p < *1e-8 and fold enrichment >4) of ONECUT2 in A549 cells. Motif analysis showed a significant enrichment of hexanucleotide ATC(G/A)AT in the vicinity of the high confidence peaks **(**Fig. 5A**)**. ATC(G/A)AT has been reported to be the binding motif for ONECUT1 and ONECUT2^21,22^, validating the efficacy and specificity of our assay.

We assigned ONECUT2 peaks to genes by using GREAT (v2.0). We found that a significant portion of peaks were located more than 50 kb away from transcription start sites (TSS) **(**Fig. 5B**)**. We defined a gene as an ONECUT2-activated or ONECUT2-repressed target when it was associated with at least one high confident ONECUT2 binding peak within 100 kb around its TSS and when the gene was significantly upregulated or downregulated (log_2_ of fold change (FC) >2 or <−2). We found 301 activated and 107 repressed target genes.

Next, we annotated the chromatin states around TSS of ONECUT2 target genes, by using the chromatin state model of A549 cells from ENCODE and Roadmap. We found that a significant portion (37.6%) of the TSS of activated target genes but not those of repressed genes (10.1%) showed chromatin marks typical of bivalent chromatin domains (Biv) and Polycomb-repressed regions (ReprPC) **(**Fig. 5C**)**. Bivalent chromatin regions are characterized by H3K4me3 and H3K27me3 at the same location, where Polycomb-repressed regions are marked mainly with repressive H3K27me3 modification. At the peak level, activating peaks were enriched for Polycomb-repressed regions (ReprPC).

Given the evidence that ONECUT2 binding sites were preferentially associated with H3K27me3 modification and ONECUT2 activated genes overlapped with PRC2 targets **(**Fig. 4B**)**, We speculated that ONECUT2 might activate gene expression by modulating the balance of H3K4me3 and H3K27me3 around the TSS of activated target genes. Thus, we profiled H3K4me3 and H3K27me3 modifications in A549-ONECUT2 and A549-ONECUT2(ΔDBD) cells by ChIP-Seq. We found a significant gain of H3K4me3 and loss of H3K27me3 around activating ONECUT2 peaks and TSS of activated targets, and no alterations in either modification around repressive ONECUT2 peaks, **(**Fig. 5D–G**)**. TSS of ONECUT2 activated target genes were marked with ONECUT2 binding, loss of H3K27me3 and gain of H3K4me3 **(**Fig. 5H**)**. These results suggest that ONECUT2 activates gene expression by increasing H3K4me3 and decreasing H3K27me3 around the TSS of its targets.

### ONECUT2 activates gene expression by modulating PRC2 occupancy

**Figure 6:**
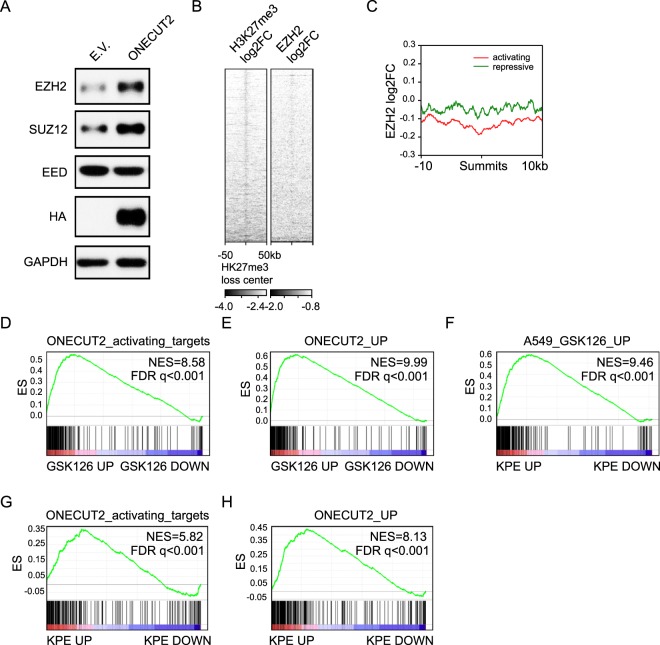
ONECUT2 modulates histone methylation through PRC2. (**A**) Western Blot of whole cell lysates from A549-ONECUT2 and A549-E.V. cells using indicated antibodies. The full-length blot is presented in Supplementary Fig. 5. (**B**) Heatmap of H3K27me3 and EZH2 log_2_ fold change signal (A549-ONECUT2 versus ΔDBD) around centers of H3K27me3 loss regions. (**C**) Plot of average EZH2 log_2_ fold change by ChIP-Seq (A549-ONECUT2 versus ΔDBD) around activating and repressive ONECUT2 peaks. (**D,E**) GSEA of ONECUT2-activated genes (**D**) and ONECUT2-upregulated genes (**E**) on gene list pre-ranked by log_2_FC after GSK126 treatment. (**F**) GSEA of GSK126 upregulation gene set on gene list pre-ranked based on log_2_FC (KPE versus KP) (Kras^G12D/+^; Trp53^fl/fl^; Eed^fl/fl^ versus Kras^G12D/+^; Trp53^fl/fl^). (**G,H**) GSEA of ONECUT2-activated genes(**G**) and ONECUT2-upregulated genes(H) on gene list pre-ranked by log_2_FC (KPE versus KE).

We next set out to determine the epigenetic machinery responsible for the changes in histone modification around ONECUT2-activated targets. Repressive H3K27me3 mark is maintained by PRC2 complex. We found EZH2 and SUZ12, two core components of PRC2, was upregulated upon ONECUT2 overexpression. EED, another component of the complex, was slightly downregulated **(**Fig. 6A**)**. While abundance of PRC2 components were altered, the overall H3k27me3 level was not changed (data not shown), indicating a site-specific rather than systemic chromatin modification change.

We profiled EZH2 occupancy in A549-ONECUT2 and A549-ONECUT2(ΔDBD) cells by ChIP-Seq. As expected, regions of H3K27me3 loss were also marked with EZH2 loss **(**Fig. 6B**)**. We observed loss of EZH2 occupancy around activating ONECUT2 peaks **(**Fig. 6C**)**, indicating ONECUT2 regulated H3K27me3 modification by modulating PRC2 occupancy.

We modulated PRC2 activity by treating A549 cells with GSK126 (5 µM for 5 days), a specific inhibitor of the EZH2. We found that genes upregulated by GSK126 were significantly overrepresented by ONECUT2-activated targets or ONECUT2-upregulated genes **(**Fig. 6D,E**)**. Importantly, genes upregulated by GSK126 treatment significantly overlapped with genes upregulated by the genetic ablation of Eed in a mouse model of *Kras*-driven lung adenocarcinoma^9^ **(**Fig. 6F**)**. Similarly, genes upregulated by Eed loss in *Kras* activated and *Tp53* deleted cells were significantly overrepresented by ONECUT2-activated targets or ONECUT2-upregulated genes **(**Fig. 6G,H**)**. These data strongly support the context-dependent oncogenicity of PRC2 and ONECUT2 in lung adenocarcinoma.

## Discussion

In this study, we reveal a context-dependent oncogenic role and epigenetic basis for the transcriptional regulation of ONECUT2 in lung adenocarcinoma. To the best of our knowledge, our work is the first systematic investigation of ONECUT2 in lung cancer progression.

ONECUT2 has early been implicated in EMT and cancer cell invasion in colorectal cancer^23^. Recent studies revealed ONECUT2 is a driver of neuroendocrine prostate cancer and a targetable master regulator of lethal prostate cancer^18,19^. However, High level of heterogeneity of lung cancer put the investigation of a new oncogene in a much more complicated situation. ASCL1 is a master regulator for neuroendocrine lung cancer^24^, but not for neuroendocrine prostate cancer. The fact that ASCL1 and ONECUT2 are both upregulated in Rb1/TP53 knockout mice and high correlation of their expression in neuroendocrine lung cancer make it difficult to discern their functional relationship without complex genetic mouse models. However, in lung adenocarcinoma, ASCL1 expression seldom overlaps with ONECUT2. After in-depth investigation, we found ONECUT2, but not ASCL1, was a prognostic gene in lung adenocarcinoma with both RAS activated and TP53 loss of function. RAS signaling pathway is the most frequently activated oncogenic pathway in lung adenocarcinoma, accounting for over one thirds of patients. Given the previous findings that Eed loss promoted lung adenocarcinoma progression and was associated with poor prognosis in KRas^G12D/+^; Trp53^fl/fl^, but not in KRas^G12D/+^ mice, and the potential regulation of PRC2 by ONECUT2 revealed by our study, we believe ONECUT2 is one of the genetic programs that confer PRC2 tumor-suppressive roles in RAS-driven lung cancer. Moreover, since PRC2 binds to chromatin without sequence specificity, our data indicated a site-specific modulation of PRC2 by ONECUT2. The context-dependent oncogenic role of ONECUT2 should be further investigated using genetic engineered mouse models.

Our transcriptome and epigenetic analysis both indicated ONECUT2 activated lineage-specific genes which correlated with ONECUT2’s tissue specificity. Of note, Nkx2-1 haploinsufficiency promotes mucinous LUAD and activation of enteric lineage markers that are normally repressed in alveolar cells^6^. *Eed* and *Trp53* loss, together with Kras activation, collectively promote formation of an aggressive mucinous adenocarcinoma^9^. Given the significant overlap of genes upregulated by *Eed* loss in the context of Kras activation and Trp53 inactivation and genes upregulated by ONECUT2 overexpression in A549 cells, we speculate that ONECUT2 overexpression induces aberrant mucinous differentiation, which, in combination with KRAS and TP53 mutations, promotes aggressive behaviors of lung adenocarcinoma. This speculation warrants further studies.

One limitation of our study is the use of single cell line and lack of loss of function study. We chose A549 as a model cell line to investigate the role of ONECUT2. We found that overexpression of ONECUT2 in immortalized human bronchial epithelial cell did not result in growth advantage or increased invasion (data not shown). We have also tested overexpression in other RAS-driven lung cancer cell lines. Of all, A549 is the only cell line which can be efficiently transdifferentiated. This implies a high level of heterogeneity in terms of genetic context and barrier to trans-differentiation in lung cancer cell lines. Unfortunately, we could not find a suitable cell line with both oncogenic KRAS mutation and intrinsically high ONECUT2 expression to carry out loss of function study. Further studies should validate the context-dependent oncogenicity of ONECUT2 in a broader system.

In summary, our study demonstrates ONCUT2 promotes RAS-driven lung cancer progression by modulating PRC2 occupancy. ONECUT2 may be an important therapeutic target in lung adenocarcinoma.

## Materials and Methods

### Cell line culture and reagents

A549 cells were obtained from the ATCC in 2012. A549 and human embryonic kidney HEK293T cells were cultured in Dulbecco’s Modified Eagle’s Medium (DMEM, Invitrogen) containing 10% fetal bovine serum (FBS, Gibco) supplemented with penicillin (100 U/ml) and streptomycin (100 mg/ml) (Invitrogen). For bioluminescence imaging, A549 cells were transduced with a fusion protein constructs encoding firefly luciferase. GSK126 were purchased from Selleck.

### Plasmids and primers

Human ONECUT2 cDNA was amplified from a mix of human lung cancer cell line cDNA with primers 5′-GAAGATCTAAGGCTGCCTACACCG-3′ (forward) and 5′-CCCTCGAGTCATGCTTTGGTACACGTG-3′ (reverse), and was cloned into pMIP-3 × HA (modified from pMIG, in which GFP was substituted with a puromycin resistance gene and 3 tandem repeats of the Hemagglutinin(HA) coding sequence were added at the N-terminus). ONECUT2(ΔDBD) was subcloned from ONECUT2 cDNA by using the reverse primer 5′-CCCTCGAGTCACGTGGCCACCTGCGAG-3′ into pMIP-3XHA. All sequences were verified by Sanger sequencing.

### Retrovirus-mediated gene expression

The pMIP retroviral vectors, together with help vectors were transfected to 293FT cells for viral packaging. 72 h after transfection, viral supernatant was collected from transfected packaging cells and concentrated using Polyethylene Glycol (PEG8000). Target cells were infected in the presence of 8 µg/ml polybrene (Sigma) and selected with 1 µg/ml puromycin (Sigma).

### Proliferation assay

Cell proliferation assays were performed using the CellTiter 96 AQueous One Solution Cell Proliferation Assay System (G3582, Promega) according to the manufacturer’s instructions.

### Soft agar colony formation assay

Cells (5 × 10^3^) were suspended in 1.5 ml of 0.35% low melting point agarose (Sigma) in DMEM containing 10% FBS, and this suspension was overlaid on pre-solidified 0.5% agarose in the same medium in ultra-low 6-well plates (Invitrogen). Normal growth medium was gently layered over the cultures every 4 days for 14 days. The colonies were stained with 0.005% crystal violet for 1 h at room temperature and counted under an inverted microscope.

### Two-chamber transwell assay

Serum-starved A549 cells (2.5 × 10^4^) were re-suspended in serum-free medium and seeded in the upper chamber of inserts (Costar) with 8 μm pore size coated with 0.2 mg/mL Matrigel (BD). FBS was used in the bottom chamber as the attractant. After 12–16 h, the cells in the upper chamber were removed using a cotton swab, and the invading cells was stained with crystal violet and counted.

### Adhesion assay

For adhesion assay, 96-well plates were coated with 5 μg/cm^2^ fibronectin (Santa Cruz), Collagen I (354231, BD), Collagen IV (354233, BD), and Matrigel (354234, BD) at room temperature for 1 h, and blocked with 10 mg/ml BSA at room temp for 1 h. A549 cells (2 × 10^4^) were seeded for 15 mins. Then, the plates were shaken for 15 s, and the detached cells were aspirated. The remaining cells were fixed, stained with crystal violet, and lysed with 2% SDS. Absorbance were measured at the wavelength of 562 nm using a microplate reader (Thermo Fisher Scientific).

### Wound healing assay

Cells were seeded in 6-well plates. When the cells reached 90% confluence, they were starved in DMEM containing 1% FBS. The cell monolayer was scraped with a 200 μl pipette tip from the center of each well and marked at the injury line. Photomicrographs of the wounds were taken at 0, 22, and 42 h.

### Immunoblotting

Whole cell lysates were prepared using RIPA buffer (Cell Signaling). Proteins were separated by 10% SDS/polyacrylamide gel electrophoresis and electro-transferred to PVDF membranes (Millipore). The membrane was blocked for 1 hour at room temperature (RT) then incubated with a primary antibody overnight at 4 °C, followed by incubation with a horseradish peroxidase-conjugated secondary antibody (Bio-rad) for 1 hour at RT. Proteins were detected by enhanced chemiluminescence (Millipore). Primary antibodies against EZH2 (5346 S, Cell Signaling), SUZ12 (3737 S, Cell Signaling), EED (05-1320, Millipore), HA (2367 S, Cell Signaling), GAPDH (G8795, Sigma) and α-tubulin (T5168, Sigma) were used in the study.

### Tissue microarray

The human lung adenocarcinoma tissue microarray HLug-Ade150Sur-01 was stained at the National Engineering Center for Biochips at Shanghai, China (http://en.superchip.com.cn/webE/index.asp) using an anti-ONECUT2 antibody (HPA057058, Sigma) at a 1:100 dilution.

### Mice

Male Balb/c nude mice aged 4–6 weeks were used in all studies. Mice were purchased from Shanghai Laboratory Animal Center, Chinese Academy of Sciences. All mice were maintained in pathogen-free conditions. All animal experiments were performed according to the guidelines for the care and use of laboratory animals and were approved by the institutional biomedical research ethics committee of Shanghai Institutes for Biological Sciences.

### Xenograft tumorigenesis

A549-ONECUT2, A549-ONECUT2(ΔDBD) or A549-E.V. cells (1 × 10^6^) in 100 µl PBS were subcutaneously inoculated at the base of the left forelimbs of 6-week-old BALB/c nude mice. Tumor diameters were measured every 3–4 days with digital calipers, and the tumor volume was calculated by the formula: Volume = (width)^2^ × length/2. On day 43, the mice were sacrificed, and tumors were isolated for weighing and imaging.

### *In vivo* bone metastasis

A549-ONECUT2, A549-ONECUT2 (ΔDBD) or A549-E.V. cells (2 × 10^5^) in PBS were injected into the left ventricle of 6-week-old male Balb/c nude mice to study metastatic activity. BLI was acquired with a NightOWL II LB 983 Imaging System (Berthold) every week. Bone damage was detected by X-ray radiography. The mice were anesthetized, arranged in a prone position on single-wrapped films (X-OMAT Kodak) and exposed for 180 s at 24 kV with a Faxitron instrument (Faxitron Bioptics). *In vivo* micro-computed tomography (micro-CT) images were obtained using a Skyscan-1076 micro-CT scanner (Skyscan) while the animals were anesthetized. The micro-CT scanner was operated at 55 kV and 181 μA with a 0.5 mm Al filter and a scan resolution of 17.4 μm. The cross-sections were reconstructed using NRecon software (Skyscan).

### Histological analysis

Hindlimb long bones of mice were excised, fixed in 10% neutral-buffered formalin for 24 hours, decalcified (10% EDTA, 2 weeks), dehydrated through a graded alcohol series, embedded in paraffin, and stained with hematoxylin and eosin.

### Definition of RAS-driven and RAS-activated ADC

TCGA-LUAD data was obtained from GDC Data Portal. Oncogenic mutations and copy number variations in the RTK/RAS/BRAF pathway were annotated using oncoKB^25^. RAS-driven ADC is defined as samples bear oncogenic alterations in KRAS, NRAS, HRAS, RIT1 or NF1. RAS-activated ADC is defined as samples bear oncogenic alterations in EGFR, ERBB2, MET, KRAS, NRAS, HRAS, RIT1, NF1, BRAF or MAP2K1.

### RNA sequencing

RNA was extracted from cells by using RNeasy Plus Mini Kit (Qiagen) according to the manufacturer’s instructions. RNA-Seq libraries were constructed using pair-end adapters with an Illumina mRNA sequencing kit. The libraries were sequenced on Illumina HiSeq 2000 or HiSeq 2500 platform.

### Chromatin immunoprecipitation sequencing

ChIP was performed using SimpleChIP Enzymatic Chromatin IP Kit (9003, Cell Signaling) following manufacturer’s instructions. The antibodies used were as follows: anti-H3K4me3 (9751S, Cell Signaling), anti- H3K27me3 (9733S, Cell Signaling), anti-EZH2 (5246S, Cell Signaling) and anti-HA (ab9110, Abcam, for ONECUT2 ChIP). ChIP DNA was used to generate sequencing libraries according to the manufacturer’s instructions for Illumina ChIP-Seq. The libraries were sequenced on Illumina HiSeq2000 or HiSeq2500 platform.

### High-throughput sequencing data analysis

RNA-Seq fastq files were trimmed to remove adapter sequences and low-quality bases with Trimmomatic^26^, and then mapped to the hg19 genome and transcriptome with Tophat2^27^ or Hisat2^28^. Raw count tables were generated using featureCounts^29^. FPKM and differential expression analyses were performed using gfold^30^ or DESeq2^31^. ChIP-Seq fastq files were trimmed to remove adapter sequences and low-quality bases and then mapped to the hg19 genome with Bowtie2^32^ or Hisat2^28^. Peak calling was performed and a bigwig coverage file for ONECUT2 was generated with MACS2^33^. Motif discovery was done with HOMER^34^. The differential binding signal tracks were generated by using deepTools^35^.

### Hierarchy clustering and heatmap

Hierarchy Clustering were performed with Cluster 3.0. The Heatmap was displayed with Java Treeview.

### Statistical analysis

Unless otherwise indicated, the data in the Figures are presented as the mean ± SD. Two-tailed Student’s t-test was performed to compare *in vitro* data. Two-sided Wilcoxon rank test was used to compare BLI data at each time point. Tumor growth and BLI curves were compared by using ANOVA. Log-rank test was used to compare animal and patient survival. A P-value less than 0.05 was considered significant.

## Supplementary information

Supplementary Figures

Supplementary Table 1

Supplementary Table 2

## Data Availability

RNA-Seq and ChIP-Seq data have been deposited in the NCBI Gene Expression Omnibus (GEO) database under Accession Number GSE102599.
