# Supplementary material for: ONECUT2 overexpression promotes RAS-driven lung adenocarcinoma progression
Source: Sci Rep. 2019 Dec 27;9:20021. doi: 10.1038/s41598-019-56277-2 (PMC6934839; doi:10.1038/s41598-019-56277-2)
Supplement: Supplementary file 1 — Supplementary Figures [file 41598_2019_56277_MOESM1_ESM.doc]

**ONECUT2 overexpression promotes RAS-driven lung adenocarcinoma progression**

Qingyang Ma, Kai Wu, Hui Li, Huichun Li, Yufei Zhu, Guohong Hu, Landian Hu and Xiangyin Kong

**Supplementary Figures**

**
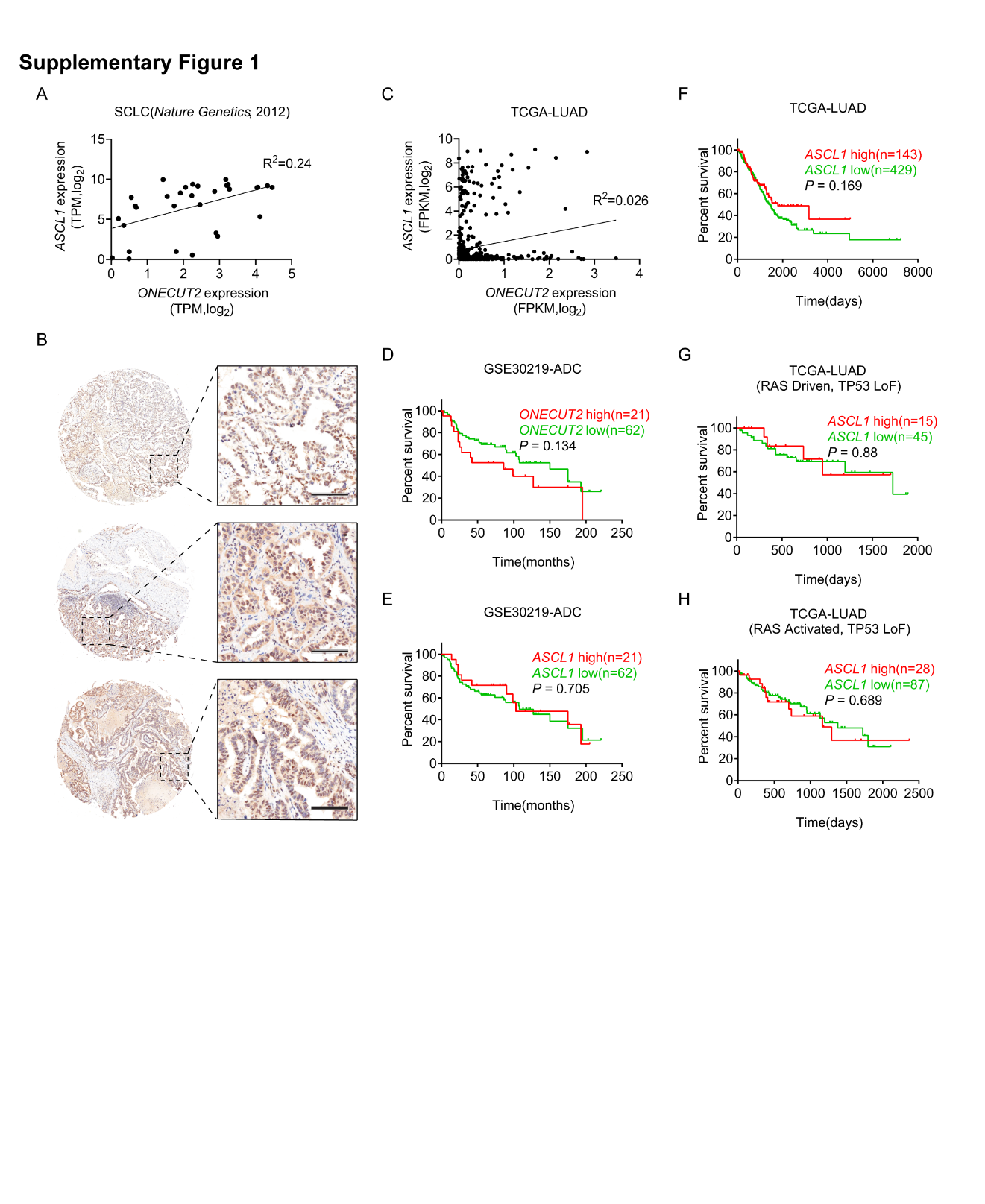
**

**Supplementary Figure 1.** (A) Scatter plot of ASCL1 and ONECUT2 expression of SCLC samples from EGAD00001000223. (B)Three representative lung adenocarcinoma tissue sections from tissue microarray, stained with ONECUT2 antibody (HPA). Scale bar: 100 µm. (C) Scatter plot of ASCL1 and ONECUT2 expression of ADC samples from TCGA-LUAD. (D-E) Overall survival analysis of ADC patients from GSE30219 stratified by the upper quantile expression level of ONECUT2 (D) or ASCL1 (E). (F) Overall survival analysis of ADC patients from TCGA-LUAD stratified by the upper quantile expression level of ASCL1. (G-H) Overall survival analysis of RAS-driven (G) ADC patients or patients with oncogenic alterations in RTK/RAS/BRAF pathway (H) in the presence of TP53 loss of function alterations from TCGA-LUAD stratified by upper quantile expression level of ASCL1.

**
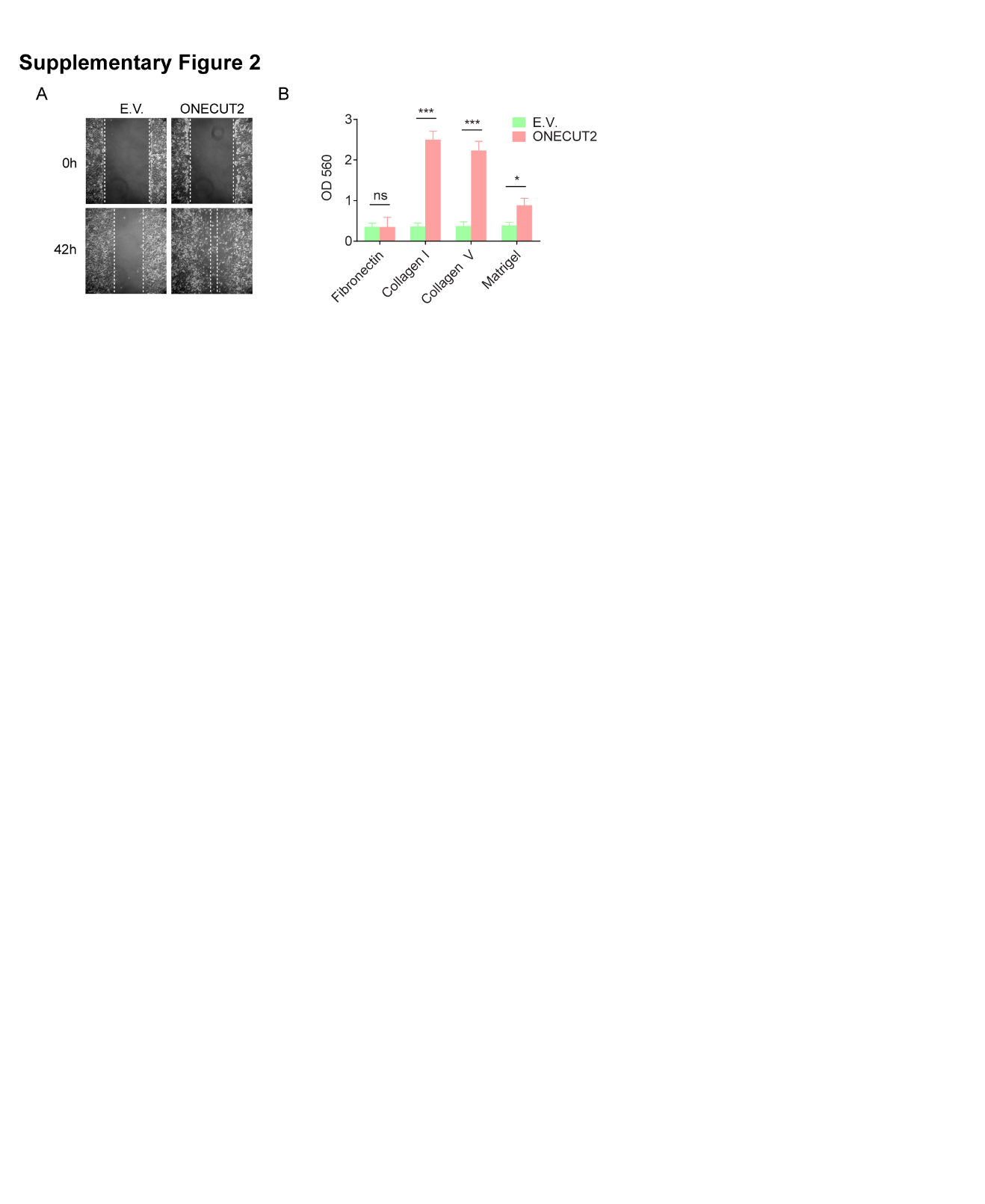
**

**Supplementary Figure 2.** (A) Would healing assay of A549-ONECUT2 and A549-E.V. cells. (B) Adhesion of A549-ONECUT2 and A549-E.V. cells. Error bars indicate mean ± SD (*p<0.01, ***p<0.001, ns: not significant, Student’s t-test).

**
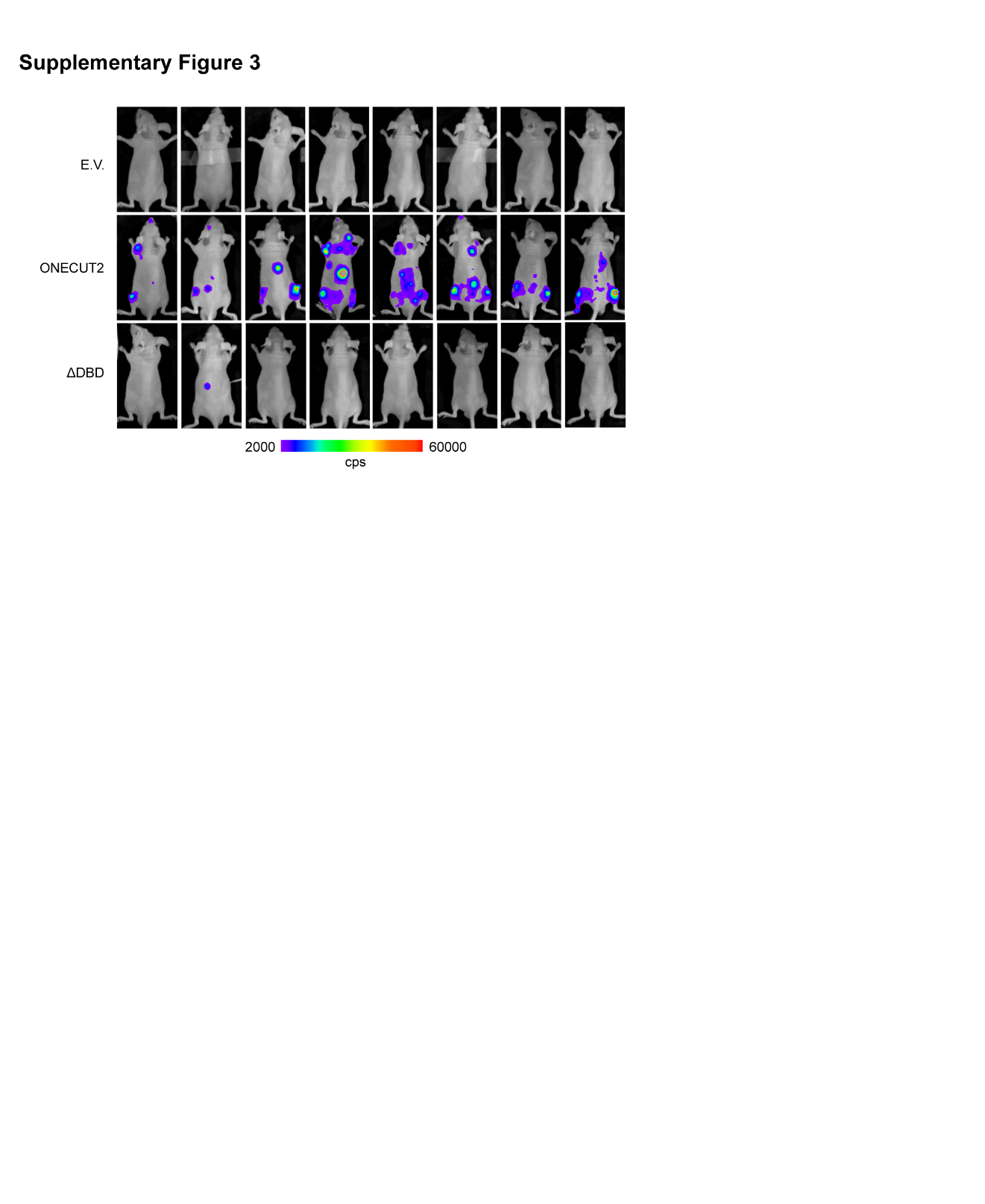
Supplementary Figure 3.** Bioluminescence imaging (BLI) of bone metastasis burden of mice implanted with A549-ONECUT2, A549-E.V. and A549-ONECUT2(ΔDBD) cells.

**
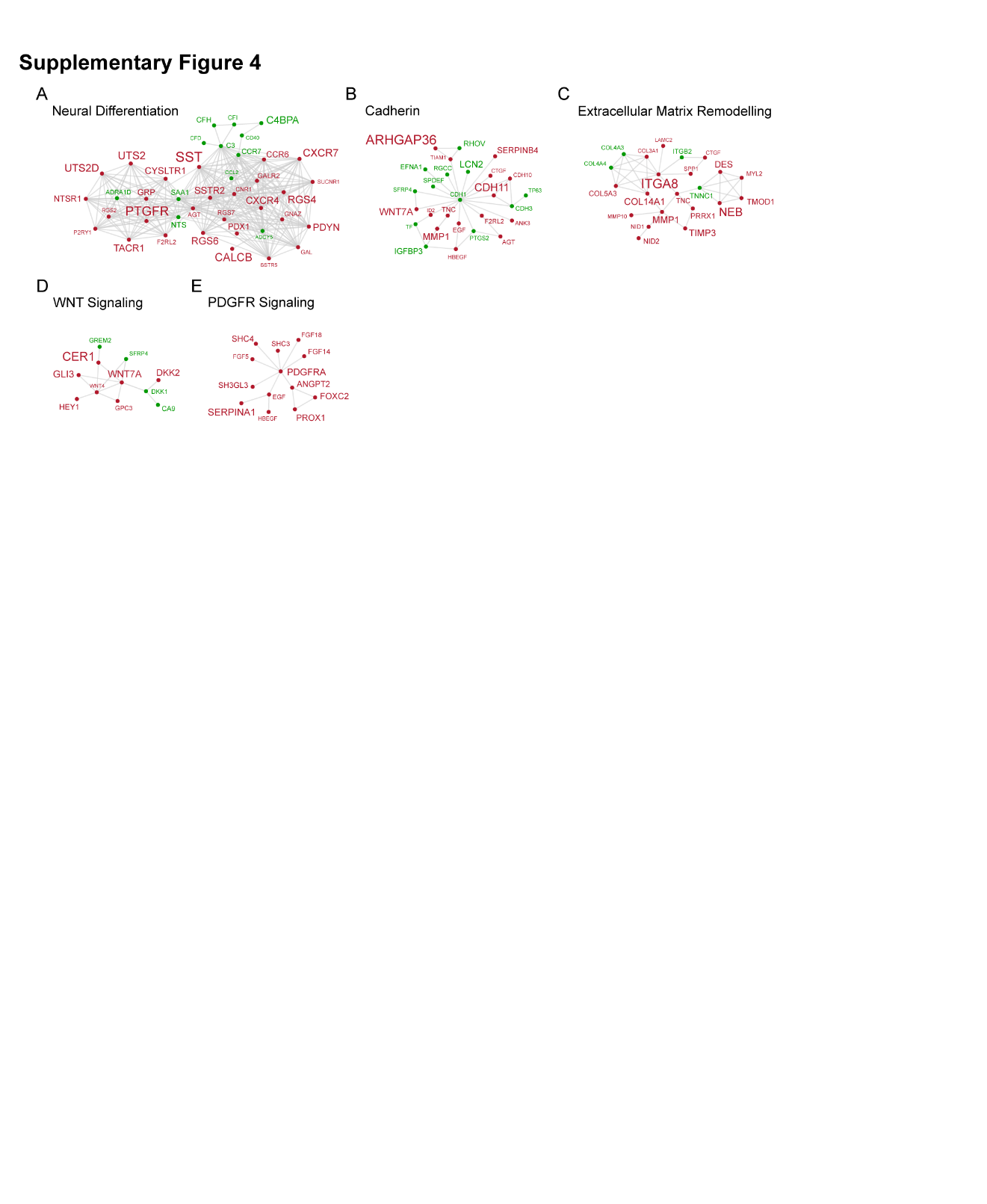
**

**Supplementary Figure 4.** (A-E) Selected subnetworks adapted from protein-protein interaction network of DEGs by STRING database. Red nodes and labels represent upregulated genes, and green nodes and labels represent downregulated genes. Label size is proportional to absolute log_2_FC (ONECUT2 versus ΔDBD).

**
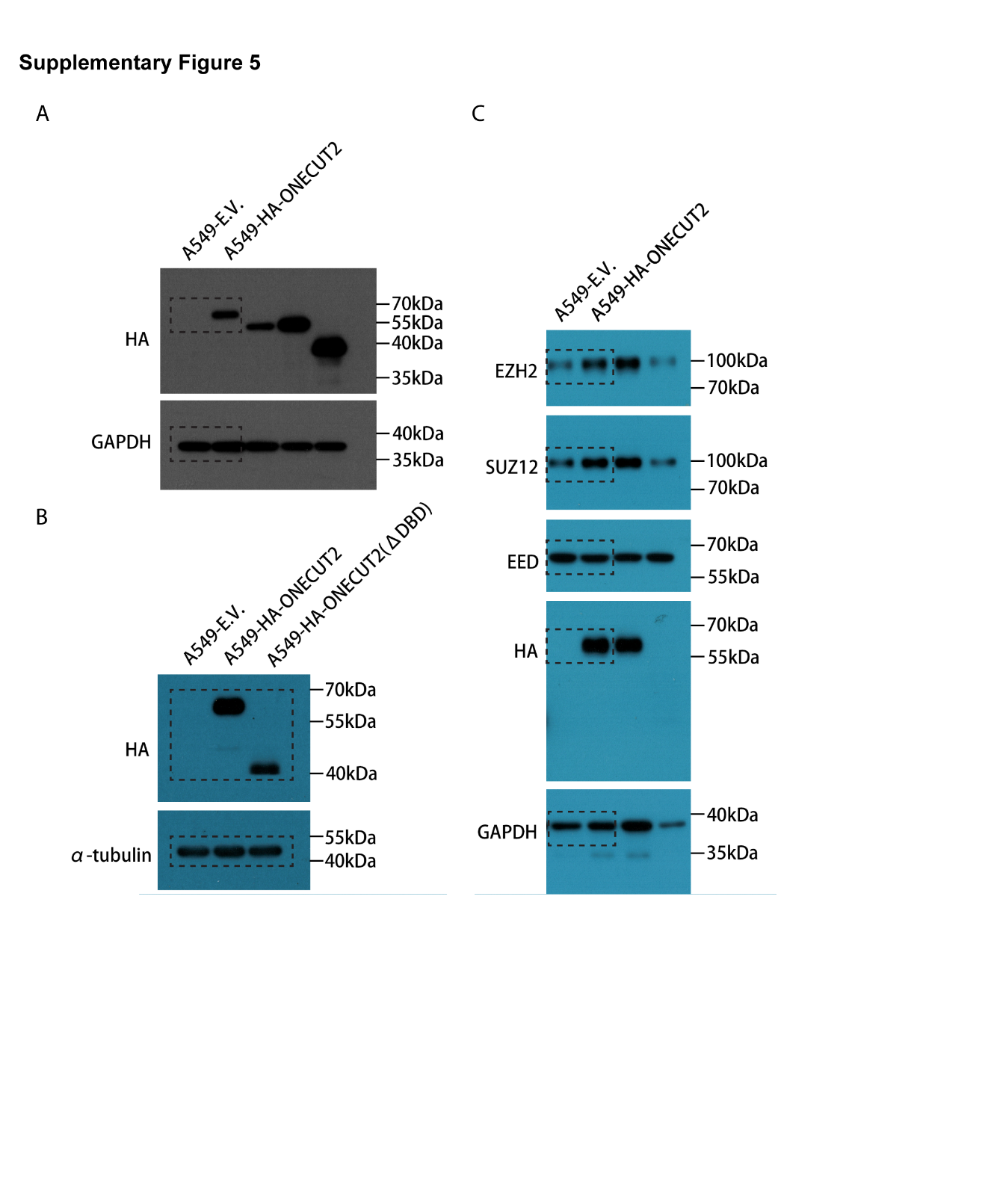
Supplementary Figure 5.** The full-length western blots for Figure 2A(A), Figure 3B(B) and Figure 6A(C).
